# Peak width of skeletonized mean diffusivity mediates the relationship between cerebral small vessel disease burden and cognitive impairment in community-dwelling older adults

**DOI:** 10.1093/braincomms/fcaf233

**Published:** 2025-06-10

**Authors:** Qili Hu, Zhenxu Xiao, Xiaowen Zhou, Qianhua Zhao, Ding Ding, Jun Zhang

**Affiliations:** Department of Radiology, State Key Laboratory of Brain Function and Disorders, Huashan Hospital, Shanghai 200040, China; Institute of Neurology, Huashan Hospital, Fudan University, Shanghai 200040, China; National Clinical Research Center for Aging and Medicine, Huashan Hospital, Fudan University, Shanghai 200040, China; National Center for Neurological Disorders, Huashan Hospital, Fudan University, Shanghai 200040, China; Institute of Neurology, Huashan Hospital, Fudan University, Shanghai 200040, China; National Clinical Research Center for Aging and Medicine, Huashan Hospital, Fudan University, Shanghai 200040, China; National Center for Neurological Disorders, Huashan Hospital, Fudan University, Shanghai 200040, China; Institute of Neurology, Huashan Hospital, Fudan University, Shanghai 200040, China; National Clinical Research Center for Aging and Medicine, Huashan Hospital, Fudan University, Shanghai 200040, China; National Center for Neurological Disorders, Huashan Hospital, Fudan University, Shanghai 200040, China; Institute of Neurology, Huashan Hospital, Fudan University, Shanghai 200040, China; National Clinical Research Center for Aging and Medicine, Huashan Hospital, Fudan University, Shanghai 200040, China; National Center for Neurological Disorders, Huashan Hospital, Fudan University, Shanghai 200040, China; Department of Radiology, State Key Laboratory of Brain Function and Disorders, Huashan Hospital, Shanghai 200040, China; National Clinical Research Center for Aging and Medicine, Huashan Hospital, Fudan University, Shanghai 200040, China; National Center for Neurological Disorders, Huashan Hospital, Fudan University, Shanghai 200040, China

**Keywords:** cerebral small vessel disease, diffusion tensor imaging, peak width of skeletonized mean diffusivity, cognition, white matter

## Abstract

Peak width of skeletonized mean diffusivity (PSMD) is a reliable marker of white matter (WM) integrity in cerebral small vessel disease (CSVD). However, the mediating role of WM damage (as measured by PSMD) in the association between CSVD and cognitive impairment remains unclear. This research applied multivariate regression analysis to explore correlations among CSVD markers, PSMD and cognitive function in older community residents. Additionally, we analysed associations between PSMD and cognitive performance across different CSVD burden levels. Simple and chain mediation analyses were employed to evaluate PSMD’s mediating role in the relationship among cerebrovascular risk factors, CSVD and cognitive decline. The cohort consisted of 273 participants categorized by CSVD severity into mild, moderate and severe groups. Each CSVD marker, along with PSMD, was significantly correlated with cognitive performance when controlling for demographic factors (age, sex and education) and apolipoprotein E (APOE) genotype. PSMD fully mediated the relationship between enlarged perivascular spaces and both the Mini-Mental State Examination and Modified Common Objects Sorting Test scores, while it partially mediated the relationships involving the CSVD burden, white matter hyperintensity volume, lacunes and cerebral microbleeds (indirect effect *P* < 0.05). Exploratory chain analysis revealed that hypertension and coronary heart disease indirectly influenced cognition through CSVD and PSMD pathways (*P* < 0.05). CSVD may impact cognitive function in older adults through the aggravated WM injury measured by PSMD. Vascular risk factors may serve as upstream determinants in the pathway linking CSVD, PSMD and cognitive dysfunction.

## Teaser

Qili Hu *et al*. report that cerebral small vessel disease could impact the cognitive function in older adults through the aggravated white matter injury measured by peak width of skeletonized mean diffusivity. Vascular risk factors, specifically hypertension and heart disease, may act as upstream determinants in the pathway.

## Graphical Abstract

**Graphical Abstract:**
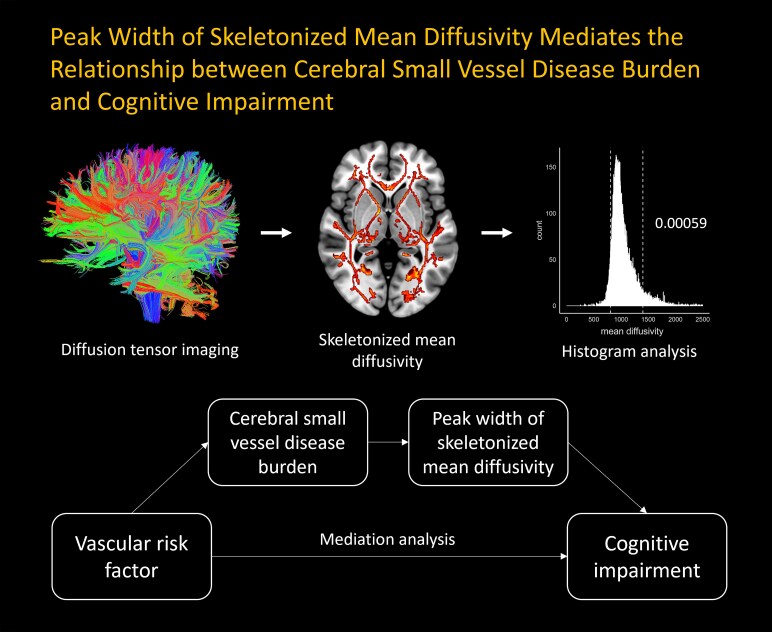

## Introduction

Cerebral small vessel disease (CSVD) is a highly prevalent and diverse condition affecting the brain’s small blood vessels in leptomeningeal and intraparenchymal regions, originating from subarachnoid or major intraparenchymal arterial circulations.^1,2^ CSVD may cause widespread ischaemic and haemorrhagic damage, and further vascular cognitive impairment and dementia.^3^ Key risk factors for CSVD include histories of stroke/transient ischaemic attack, hypertension, head trauma, hyperlipidaemia and heart disease, which may potentially impact the brain blood flow and exacerbate the CSVD progression.^4-7^

In CSVD, diminished cerebral perfusion induces chronic hypoperfusion, initiating a pathological cascade encompassing endothelial dysfunction, blood–brain barrier disruption, perivascular neuronal degeneration and programmed cell death, driven by inflammatory responses and oxidative stress-mediated cytotoxicity.^8^ The white matter (WM) regions of the brain contain organized networks critical for neurobehavioural functions.^9^ Progressive tissue injury may ultimately lead to localized or widespread white matter pathology (demyelination and axonal degeneration), subsequently inducing cognitive decline, neuropsychiatric symptoms and motor dysfunction.^10,11^

Diffusion tensor imaging (DTI), an advanced magnetic resonance imaging (MRI) modality, enables the identification of microstructural alterations in WM.^12,13^ However, the broader application of DTI is limited by complex processing steps, such as eliminating significant cerebrospinal fluid signals from mean diffusivity images. Baykara *et al.*^14^ developed the peak width of skeletonized mean diffusivity (PSMD), a DTI-derived automated metric for evaluating WM injury. PSMD showed high test–retest repeatability against different scanners and MRI protocols.^14,15^ Furthermore, research consistently links PSMD to cognitive impairment in individuals with CSVD.^14,16-20^

Previous studies investigated the relationships among CSVD, WM injury and cognitive impairment, and demonstrated the damage to WM fibre tracts, marked by reduced FA and elevated mean diffusivity (MD). These disruptions in WM integrity correlate significantly with disease severity and cognitive dysfunction.^21^ Other studies revealed that WM injury indicated by WM network efficiency, mean WM FA and white matter hyperintensity volume (WMHV) significantly mediates the relationship between CSVD and clinical symptoms, including cognitive impairment, apathy and gait disturbances.^22-24^ In CSVD, PSMD has emerged as a promising biomarker for assessing WM integrity. However, few studies have utilized PSMD to measure WM injury and investigate its mediating role in CSVD-related cognitive impairment.

This study employed mediation analysis to test the hypothesis that CSVD causes WM injury (measured by PSMD), thereby influencing cognitive function in older adults. We also tried to illustrate the roles and potential mechanisms of vascular risk factors within this association.

## Materials and methods

### Study participants

Subjects enrolled in this study were recruited from the Shanghai Aging Study, with detailed recruitment protocols and study design described elsewhere.^25,26^ Participants who were administered the MRI scanning from 1 January 2010 to 30 April 2010 were eligible for the current study with the inclusion criteria including (i) being aged ≥55 years; (ii) being permanent registered residents in the community; (iii) not having schizophrenia or intellectual disability; (iv) being able to complete the physical and neuropsychological examinations; (v) without any contraindications to MRI scanning and (vi) having MRI images with sequences for CSVD evaluation and PSMD calculation. Demographic and lifestyle data were collected through structured questionnaires, and the apolipoprotein E (APOE) genotype was determined using standard Taqman SNP assays, with carriers of ≥1 ɛ4 allele classified as APOE ɛ4 positive.

This study was approved by Huashan Hospital’s Ethics Committee, Fudan University (2009-195 and 2022-632). Written informed consent was obtained from all participants and/or their legally acceptable guardians.

### Vascular risk factors

Neurologists from the Huashan Hospital’s Department of Neurology performed standardized clinical assessments, systematically documenting physician-confirmed diagnoses of hypertension, diabetes, hyperlipidaemia, stroke, head trauma and cardiovascular disorders (including coronary artery disease, valvulopathy, cardiomyopathies and arrhythmias). All reported medical histories were cross-verified against archived clinical documentation from participants’ healthcare providers.

### Neuropsychological assessments

A neuropsychological battery administered by trained psychometrists, including^25,26^: (i) the Mini-Mental State Examination (MMSE); (ii) the Conflicting Instructions Task; (iii) the Stick Test; (iv) the Modified Common Objects Sorting Test (MCOST); (v) the Auditory Verbal Learning Test; (vi) the Modified Fuld Object Memory Evaluation; (vii) the Trail-making Tests A and B and (viii) the Renminbi (Chinese currency) Test. In the current study, we only analysed data from Tests 1 to 4, which were completed by all participants. The Supplementary Materials contain comprehensive descriptions of these neuropsychological tests.

### MRI parameters

MRI data were acquired on a 1.5T GE scanner. The imaging protocol included: T_1_-Weighted Imaging with TR/TE = 11/5 ms, a flip angle = 15°, field of view (FOV) = 250 mm and slice thickness/gap = 1.5/1.5 mm; T_2_-Weighted Imaging with TR/TE = 4600/12 ms, FOV = 240 mm and slice thickness/gap = 4/4 mm; Fluid-Attenuated Inversion Recovery with TR/TE = 11000/155 ms, an inversion time = 2250 ms, FOV = 240 mm and slice thickness/gap = 4/4 mm; Gradient Recalled Echo T_2_*-Weighted Imaging with TR/TE = 740/16 ms, a flip angle = 20°, FOV = 240 mm and slice thickness/gap = 4/4 mm; Diffusion Weighted Imaging was performed using a single-shot echo-planar imaging technique enhanced by simultaneous multi-slice technology, with TR = 8000 ms, TE = 80 ms, employing 27 diffusion directions, a *b*-value = 1000 s/mm² and acquisition at a single *b* = 0 s/mm², FOV = 230 mm, flip angle = 90° and slice thickness/gap = 6/6 mm.

### MRI processing

PSMD calculation was performed using the toolbox (https://github.com/miac-research/psmd).^14^ The corresponding analysis code (calc_PSMD.sh) has been made available in the Supplementary Materials. All structural images underwent quality control for complete hemispheric coverage, with the manual exclusion of scans exhibiting significant motion artifacts or susceptibility distortions. The average of the root mean squared displacement relative to the previous volume, used as a measure of head motion, was calculated. Intergroup comparisons of mean root mean squared displacement relative to the previous volume demonstrated no statistically significant variation (*P* = 0.51).

The PSMD calculation was performed through a standardized computational pipeline including DTI skeletonization and histogram analysis. Initially, DTI data were skeletonized using the Tract-Based Spatial Statistics procedure (TBSS) algorithm implemented in the Functional Magnetic Resonance Imaging of the Brain (FMRIB) Software Library (FSL).^27^ Nonlinear registration to the FMRIB58_FA standard template [1 mm isotropic resolution, fractional anisotropy (FA) threshold = 0.2] was executed, followed by projection of MD maps onto the derived skeleton using FA-optimized warping parameters. Each participant’s registration results were visually inspected to ensure good alignment. Assessments were performed by overlaying the FMRIB58_FA template skeleton onto each participant’s registered image in MRIcroGL. Two independent raters evaluated whether key tracts (corpus callosum, internal capsule, corticospinal tract, etc.) were properly aligned with the FMRIB58_FA template, with clear boundaries and no visible misregistration (e.g. a given tract may be warped so far that it becomes aligned to a totally different tract in the target image). All ratings were performed blinded to clinical data, and any discrepancies were resolved by a third arbitrator. To ensure full transparency and allow for independent verification of our quality control process, we have uploaded the representative registration quality check figures to Zenodo (a general-purpose open-access repository developed under the European OpenAIRE programme; DOI: 10.5281/zenodo.15220265).

**Figure 1:**
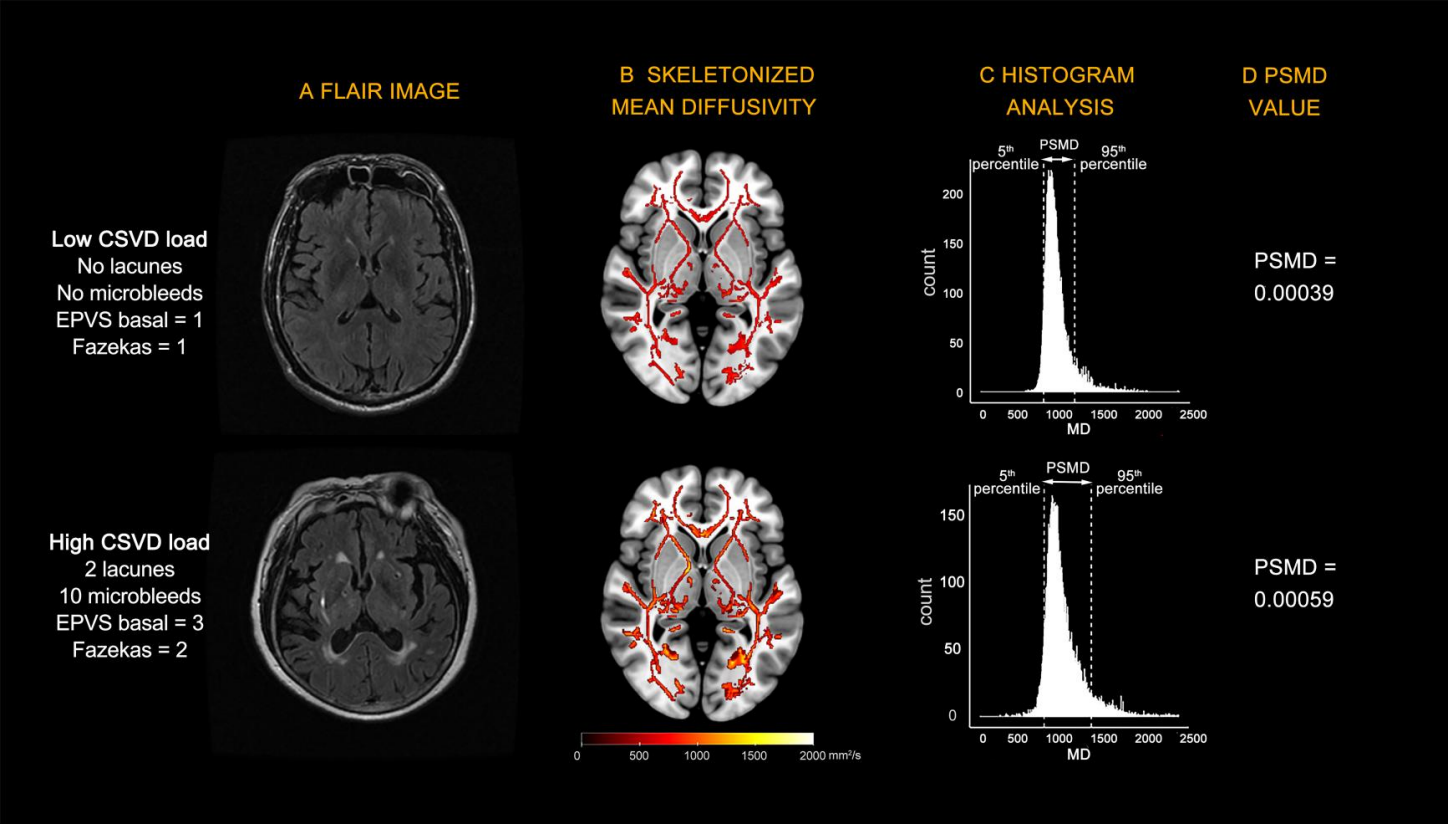
**Examples of PSMD calculation illustration from two study participants.** One with low CSVD burden (*top*) and one with high CSVD burden (*bottom*): (**A**) total CSVD burden on MRI; (**B**) skeletonized mean diffusivity maps: Individual mean diffusivity images are normalized to standard space and projected onto the skeleton template. (**C**) Histogram analysis used to obtain the PSMD value. PSMD is calculated as the difference between the 95th and 5th percentiles. (**D**) The final calculated PSMD values. EPVS, enlarged perivascular space; PSMD, peak width of skeletonized mean diffusivity; CSVD, cerebral small vessel disease.

To mitigate cerebrospinal fluid partial volume contamination, sequential masking operations were applied: (i) The default TBSS skeleton mask (FA > 0.3) eliminated peripheral white matter regions and (ii) The updated PSMD-specific exclusion mask (2019 release) removed ventricular-adjacent structures including the fornix, midbrain nuclei and superficial periventricular zones. This iterative masking reduced voxel counts from the initial TBSS-derived 212 081 to 86 406 voxels (2016 PSMD mask), and ultimately to 85 186 voxels (2019 mask) through the exclusion of 1210 predominantly midbrain and subcortical voxels. Final PSMD computation employed histogram analysis of MD values within this standardized voxel space, calculated as the arithmetic difference between the 95th and 5th percentile MD values. The calculation process of PSMD for two study participants is illustrated in Fig. 1.

### Assessment of CSVD MRI markers

CSVD imaging markers were assessed by two experienced neurologists following STRIVE consensus guidelines.^28,29^ Cerebral microbleeds (CMBs) were defined as spherical hypointensities (≤10 mm diameter) on T_2_*-MRI sequence. The number of CMBs was recorded. White matter hyperintensities (WMHs) were evaluated by both the Fazekas scale and WMHV. The WMHV was automatically segmented utilizing a deep-learning method refined on the United Imaging platform using fluid-attenuated inversion recovery images.^30^ The Fazekas scale, applied by two proficient neurologists, separately assessed periventricular and deep WMHs, with scores summing from 0 to 6.^31^ Lacunes were defined as cerebral spinal fluid-like hypointensities (3–15 mm), encircled by a T2-FLAIR hyperintense rim. Enlarged perivascular spaces (EPVSs) were assessed and categorized within the basal ganglia, with scoring as follows: 0 = no EPVS; 1 = 1–10 EPVS; 2 = 11–20 EPVS; 3 = 21–40 EPVS and 4 = more than 40 EPVS.

The composite CSVD burden score (range: 0–4) was calculated using a cumulative system incorporating five neuroimaging markers^32^: periventricular Fazekas score = 3 or deep Fazekas ≥2, presence of lacunes, presence of CMBs and basal ganglia EPVS grades 2 and 3. Based on this score, participants were stratified into three categories: mild (score 0), moderate (scores 1–2) and severe (scores 3–4) CSVD burden.

### Statistical analysis

Data analysis employed R with significance at two-tailed *P* < 0.05. The normality of continuous variables was evaluated through Kolmogorov–Smirnov tests. Normally distributed variables underwent ANOVA with *post hoc* Tukey tests; others were analysed via Kruskal–Wallis H comparison. Categorical data were presented as frequencies (%) and analysed via chi-square testing.

Linear regression analyses were performed to evaluate the associations between the CSVD markers and cognitive performance and between PSMD and cognitive performance, with age, sex, education years and APOE ɛ4 status included as covariates. Subsequently, we assessed PSMD–cognition association across different groups with mild, moderate and severe CSVD burden. To investigate a potential interaction effect between CSVD burden and PSMD on cognitive function, an interaction term was incorporated into the multivariate linear regression model.^14,16-20,33-37^ The interaction model is depicted by the equation:


$$Cognition\;scores=\;\beta_{0}+\beta_{1}\;Age+\beta_{2}Sex+\beta_{3}Edu+\beta_{4}APOE4+\beta_{5}Group+\beta_{6}PSMD+\beta_{7}(PSMD*Group)$$

Simple mediation analyses using the mediation package were applied to assess the mediation effect of PSMD on the CSVD–cognition association, controlling for age, sex and education. The mediation package is specifically designed for single mediator analysis and provides a robust bootstrap method for estimating confidence intervals of direct and indirect effects. The cognitive scales associated with both PSMD and CSVD markers in the regression analysis were incorporated into mediation models. Multicollinearity between variables was examined via variance inflation factor values in each mediation model. All variance inflation factor values for the included independent variables were less than 5, indicating no significant multicollinearity. Bootstrapping assessed the indirect effect’s significance with bias-corrected and accelerated confidence intervals. Additionally, vascular risk factors were included in an exploratory chain mediation analysis using the Lavaan package, with age and sex included as covariates. For our chain mediation analysis, we chose the lavaan package due to its flexibility and comprehensive capabilities in structural equation modelling.

## Results

### Participants characteristics

The cohort included 273 participants stratified by CSVD burden: 76 (27.8%) mild, 162 (59.3%) moderate and 35 (12.8%) severe. Mild CSVD participants were significantly younger than moderate/severe groups (*P* = 0.017), with no significant intergroup differences in sex, education years or APOE ɛ4 carrier rates.

Participants with mild CSVD were significantly younger than those with moderate or severe CSVD (*P* = 0.017). No significant intergroup differences were observed in sex, education years or the per cent of APOE ɛ4 allele carrier. Moderate/severe CSVD had elevated hypertension rates versus mild (*P* = 0.002). Severe CSVD showed elevated coronary heart disease rates relative to mild and moderate groups (*P* = 0.018). In terms of MRI characteristics, significant differences were identified across the groups concerning the percentage of participants with CMB numbers, lacune numbers and per cent of participants with moderate to severe basal ganglia EPVS, as well as WMHV and PSMD (*P* < 0.001). However, intergroup comparisons revealed no significant differences in intracranial volume. Regarding cognitive functions, significant discrepancies were found in the Item Naming score of the MCOST among the groups (*P* = 0.034). However, MMSE score, Categorization score in MCOST and Category Naming score in MCOST showed no intergroup differences (Table 1).

**Table 1:** Demographic characteristics of participants

	All	CSVD burden			
		Mild	Moderate	Severe	*P*-value
*n*	273	76	162	35	
Demographic characteristics					
Age, y, mean (SD)	68.4 (5.8)	67 (5.0)	69 (6)	70 (6.2)	**0**.**017**
Female, *n* (%)	152 (55.7)	48 (62.3)	87 (53.7)	17 (48.6)	0.260
Education, y, mean (SD)	11.5 (4.0)	11.8 (3.6)	11.3 (4.1)	11.4 (4.1)	0.750
APOE ɛ4 carrier, *n* (%)	39 (14.5)	10 (13.3)	25 (15.7)	4 (11.4)	0.763
Vascular risk factors					
BMI, mean (SD)	24.9 (3.6)	25 (4)	25 (3)	26 (4)	0.412
Hypertension, *n* (%)	149 (54.6)	30 (39.0)	93 (57.4)	26 (74.3)	**0**.**002**
Hyperlipidaemia, *n* (%)	105 (38.5)	33 (42.9)	60 (37.0)	12 (34.3)	0.553
Coronary heart disease, *n* (%)	29 (10.6)	6 (7.8)	14 (8.6)	9 (25.7)	**0**.**018**
Diabetes, *n* (%)	40 (14.7)	8 (10.4)	24 (14.8)	8 (22.9)	0.232
Smoking, *n* (%)	32 (11.7)	5 (6.5)	22 (13.6)	5 (14.3)	0.250
MRI findings					
CMBs, *n* (%)	37 (13.6)	0 (0)	15 (9.3)	22 (62.9)	**＜0**.**000**
WMHV, cm^3^, mean (SD)	11.7 (10.4)	7.2 (2.8)	10.7 (8.0)	26.1 (16.7)	**＜0**.**000**
Lacunes, *n* (%)	50 (18.3)	0 (0.0)	22 (13.6)	28 (80)	**＜0**.**000**
ICV, cm^3^, mean (SD)	1404.9 (120.5)	1395.6 (111.7)	1403.8 (127.2)	1430.1 (105.3)	0.256
EPVS, *n* (%)	65 (23.8)	0 (0)	45 (27.8)	20 (57.1)	**＜0**.**000**
PSMD, mean (SD)	4.66 (1.1)	4.43 (0.9)	4.65 (1.1)	5.18 (1.3)	**0**.**011**
Neuropsychological tests					
MMSE, mean (SD)	28.4 (2.0)	28.9 (1.4)	28.3 (2.0)	28 (2.8)	0.170
MCOST, mean (SD)					
Item naming	40.5 (2.2)	41.1 (1.1)	40.3 (2.4)	40.0 (2.5)	**0**.**034**
Categorization	38.8 (6.2)	39.7 (2.8)	39.9 (6.2)	36.5 (9.8)	0.087
Category naming	5.8 (1.5)	6.0 (1.3)	5.8 (1.5)	5.4 (1.9)	0.277

Notes: Numerical data are reported as mean (standard deviation); categorical variables as *n* (%). Group comparisons employed Kruskal–Wallis (continuous) or chi-square (categorical) tests, with statistically significant findings (*P* < 0.05) bolded in tables. Abbreviations: SD, standard deviation; CSVD, cerebral small vessel disease; BMI, body mass index; EPVS, enlarged perivascular space; CMBs, cerebral microbleeds; WMHV, white matter hyperintensity volume; ICV, intracranial volume; PSMD, peak width of skeletonized mean diffusivity; MMSE, mini-mental state examination; MCOST, modified common objects sorting test.

### Correlations of PSMD, CSVD, with cognitive performance

As shown in Table 2, univariate regression analyses revealed significant negative correlations between the MMSE score and PSMD (*β* = −0.39, *P* < 0.001), CSVD burden (*β* = −0.36, *P* = 0.001), CMB numbers (*β* = −0.09, *P* < 0.001), WMHV (*β* = −0.04, *P* < 0.001) and lacune numbers (*β* = −0.45, *P* < 0.001). Similarly, the Categorization in MCOST test score was also negatively correlated with PSMD (*β* = −1.55, *P* < 0.001), CSVD burden (*β* = −1.29, *P* < 0.001), CMB numbers (*β* = −0.28, *P* < 0.001) and WMHV (*β* = −1.48, *P* < 0.001). After adjusted for age, sex, education level and APOE ɛ4 in multivariate linear regression models, PSMD (*β* = −0.28, *P* = 0.015), CMB numbers (*β* = −0.07, *P* < 0.001) and WMHV (*β* = −0.03, *P* = 0.003) remained negatively correlated with the MMSE score, while CSVD burden exhibited a marginally insignificant correlation with the MMSE score (*β* = −0.20, *P* = 0.053). Additionally, PSMD (*β* = −1.75, *P* < 0.001), CSVD burden (*β* = −0.39, *P* < 0.001), WMHV (*β* = −0.11, *P* = 0.002) and lacune numbers (*β* = −1.21, *P* < 0.001) retained significantly negative correlations with the Categorization in MCOST score. EPVS was not significantly associated with either the MMSE or Categorization in MCOST test score in univariate and multivariate regression models. The scores of the Conflicting Instructions Task and the Stick Test were not found to be significantly correlated with both CSVD characteristics and PSMD simultaneously (Supplementary Tables 1–6).

**Table 2:** Correlations between PSMD, CSVD characteristics and cognitive performance

MMSE	Univariate analysis		Multivariate analysis	
	*β* (95% CI)	*P*-value	*β* (95% CI)	*P*-value
PSMD	−0.39 (−0.61 to −0.18)	**＜0**.**001**	−0.28 (−0.50 to −0.05)	**0**.**015**
CSVD burden	−0.36 (−0.58 to −0.15)	**0**.**001**	−0.20 (−0.40 to 0.00)	0.053
CMBs	−0.09 (−0.13 to −0.05)	**＜0**.**001**	−0.07 (−0.11 to −0.04)	**＜0**.**001**
WMHV	−0.04 (−0.07 to −0.02)	**＜0**.**001**	−0.03 (−0.05 to −0.01)	**0**.**003**
Lacunes	−0.45 (−0.64 to −0.27)	**＜0**.**001**	−0.36 (−0.52 to −0.20)	**＜0**.**001**
EPVS	−0.16 (−0.42 to 0.11)	0.250	0.02 (−0.22 to 0.27)	0.853
Categorization in MCOST				
PSMD	−1.55 (−2.23 to −0.87)	**＜0**.**001**	−1.75 (−2.46 to −1.04)	**＜0**.**001**
CSVD burden	−1.29 (−1.96 to −0.61)	**＜0**.**001**	−0.89 (−1.53 to −0.25)	**0**.**007**
CMBs	−0.28 (−0.41 to −0.15)	**＜0**.**001**	−0.24 (−0.36 to −0.13)	**＜0**.**001**
WMHV	−0.13 (−0.20 to −0.06)	**＜0**.**001**	−0.11 (−0.17 to −0.04)	**0**.**002**
Lacunes	−1.48 (−2.05 to −0.91)	**＜0**.**001**	−1.21 (−1.74 to −0.68)	**＜0**.**001**
EPVS	−0.80 (−1.64 to 0.04)	0.061	−0.36 (−1.16 to 0.43)	0.367

Note: Model 1: Simple linear regression analysis. Model 2: including age, sex, education and APOE ɛ4 as covariates. *P*-values that reach statistical significance are highlighted in bold. Abbreviations: CSVD, cerebral small vessel disease; PSMD, peak width of skeletonized mean diffusivity; MMSE, mini-mental state examination; CMBs, cerebral microbleeds; WMHV, white matter hyperintensity volume; EPVS, enlarged perivascular space; MCOST, modified common objects sorting test; APOE ɛ4, apolipoprotein E ɛ4.

### Correlation between PSMD and cognition in different CSVD burden groups

**Figure 2:**
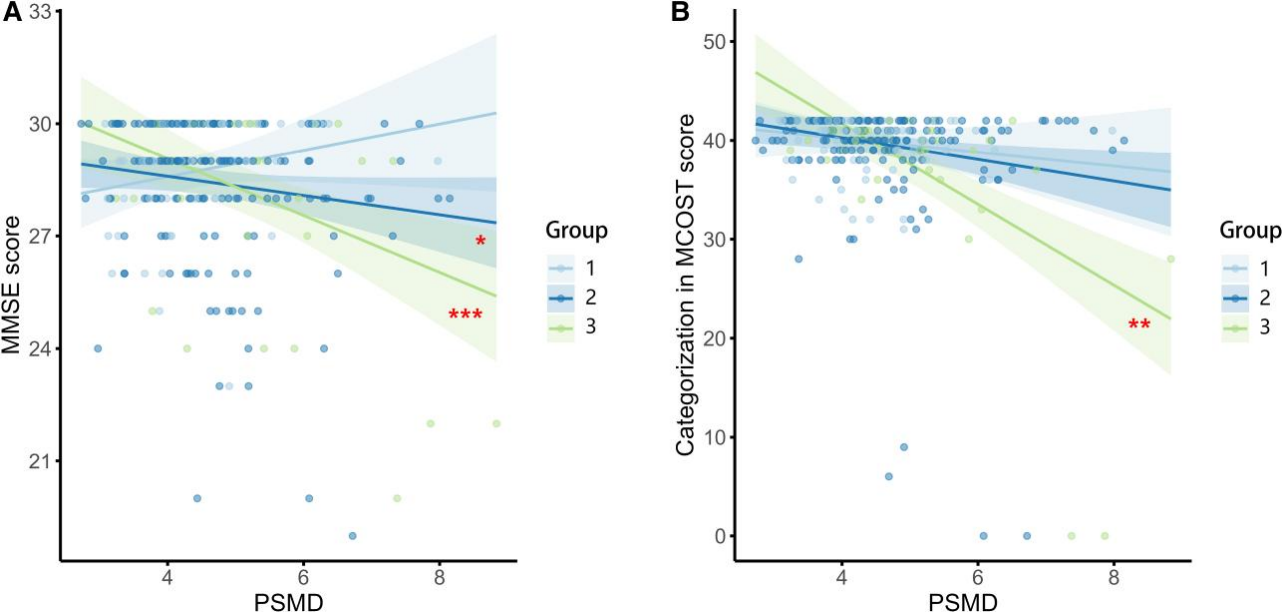
**Interactive effects of PSMD and CSVD burden on cognitive performance.** This figure depicts the results from a **multivariate linear regression analysis** investigating the interactive effect of PSMD and CSVD burden on cognitive assessments, specifically the MMSE (**A**) and MCOST scores (**B**). As the PSMD value increased, the score for Categorization in MCOST decreased more dramatically in participants with high CSVD burden compared with those with mild burden (*P* for interaction = 0.001, *n* = 267). As the PSMD value increased, the MMSE score decreased faster in the moderate burden group (*P* for interaction = 0.021, *n* = 269) or severe burden group (*P* for interaction < 0.001, *n* = 269) compared with the mild group. Adjustments were made for age, sex, educational level and APOE ɛ4 status. Group 1 represents participants with mild CSVD burden (Total score of 0); Group 2 signifies those with moderate CSVD burden (Total score of 1–2) and Group 3 indicates participants with severe CSVD burden (Total score of 3–4). Significance levels are denoted as *P < 0.05, **P < 0.01 and ***P < 0.001. PSMD, peak width of skeletonized mean diffusivity; CSVD, cerebral small vessel disease; MMSE, mini-mental state examination; MCOST, modified common objects sorting test; APOE, apolipoprotein E.

As shown in Fig. 2, significant interactions between PSMD and the CSVD burden groups on MMSE and Categorization in MCOST scores were observed. As the PSMD value increased, the score for Categorization in MCOST decreased more dramatically in participants with high CSVD burden compared with those with mild burden (*P* for interaction = 0.001). As the PSMD value increased, the MMSE score decreased faster in the moderate burden group (*P* for interaction = 0.021) or severe burden group (*P* for interaction < 0.001) compared with the mild group. These results were validated using bootstrap analysis, and the findings remained consistent (Supplementary Table 7).

### Mediating effect of PSMD in the CSVD–cognition association

**Figure 3:**
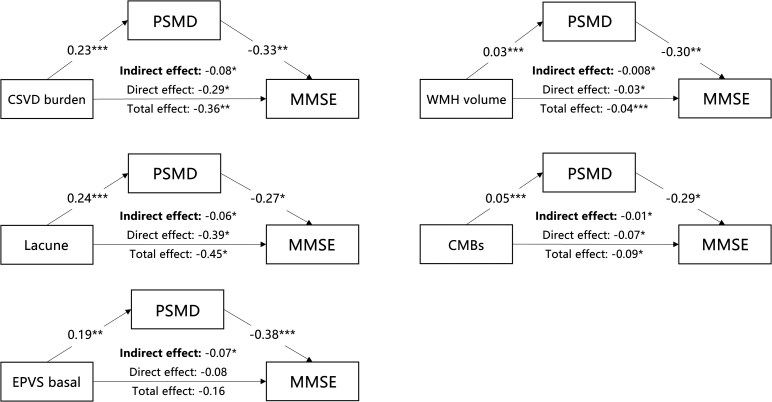
**Mediation effect of CSVD characteristics on MMSE scores via PSMD. Simple mediation analysis** illustrating the indirect effect of CSVD characteristics on MMSE scores through PSMD. The total number of participants was 273. Standardized coefficients (*β*) quantify each association, with significance levels marked as *P < 0.05, **P < 0.01 and ***P < 0.001. CSVD, cerebral small vessel disease; PSMD, peak width of skeletonized mean diffusivity; MMSE, mini-mental state examination; WMHV, white matter hyperintensity volume; CMBs, cerebral microbleeds; EPVS, enlarged perivascular space.

**Figure 4:**
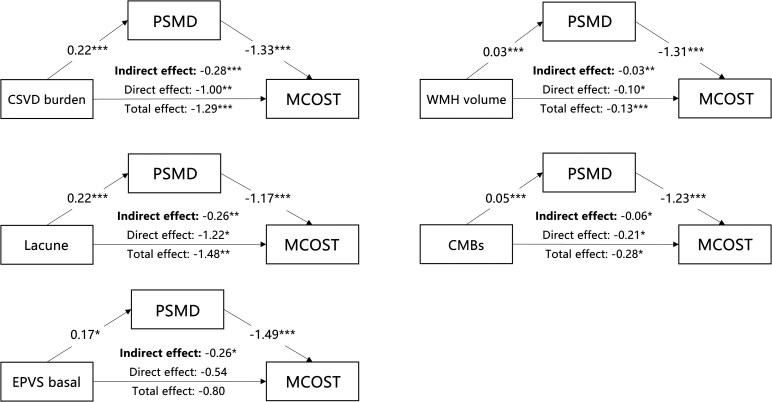
**Mediation effect of CSVD characteristics on MCOST scores via PSMD.** This figure presents the results from a **simple mediation analysis** exploring the indirect impact of CSVD characteristics on MCOST scores mediated by PSMD. The total number of participants was 271. Associations are quantified by standardized coefficients (*β*), with significance levels indicated as *P < 0.05, **P < 0.01 and ***P < 0.001. CSVD, cerebral small vessel disease; PSMD, peak width of skeletonized mean diffusivity; MMSE, mini-mental state examination; WMHV, white matter hyperintensity volume; CMBs, cerebral microbleeds; EPVS, enlarged perivascular space.

As shown in Figs 3 and 4, PSMD fully mediated the relationship between EPVS and both MMSE and MCOST scores, while it partially mediated the relationships involving the CSVD burden, WMHV, lacunes and CMBs (indirect effect *P* < 0.05). For the MMSE score, the proportions of the total effect mediated by PSMD for CSVD burden, WMHV, lacunes, CMBs and EPVS were 21, 19, 14, 16 and 47%, respectively. Regarding the Categorization score in MCOST, the proportions of the total effect mediated by PSMD for CSVD burden, WMHV, lacunes, CMBs and EPVS were 22, 26, 26, 23 and 32%, respectively.

### Impact of vascular risk factors on CSVD–PSMD–cognition pathways

**Figure 5:**
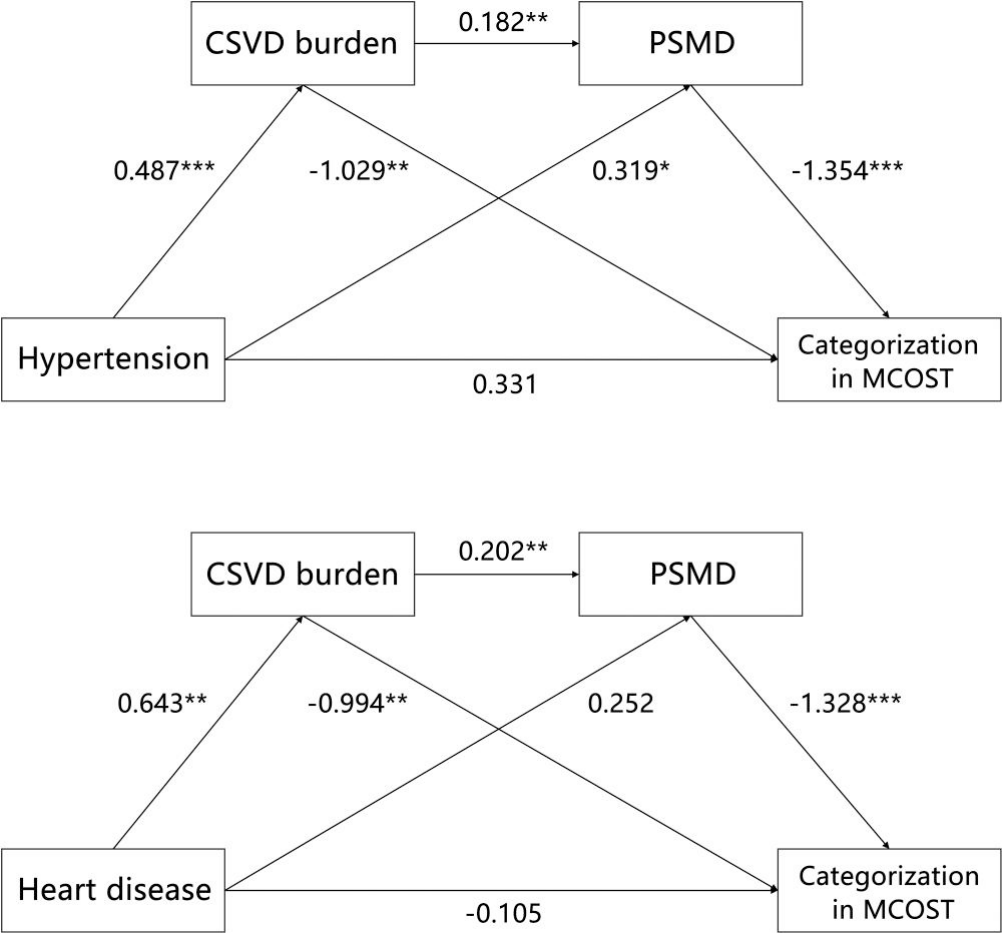
**Influence of hypertension and coronary heart disease on cognitive function via CSVD and PSMD pathways.** Illustrating findings from an exploratory chain mediation analysis using structural equation modelling to assess how hypertension and coronary heart disease indirectly affect cognitive function through CSVD and PSMD. The total number of participants was 271. Standardized coefficients (*β*) for each pathway are reported, with significance levels highlighted as *P < 0.05, **P < 0.01 and ***P < 0. 001. CSVD, cerebral small vessel disease; PSMD, peak width of skeletonized mean diffusivity; MCOST, modified common objects sorting.

Exploratory chain analysis revealed that hypertension and coronary heart disease indirectly influenced cognition via CSVD and PSMD pathways. Notably, the indirect impact of smoking, hyperlipidaemia, BMI and diabetes through CSVD and PSMD was found to be insignificant (Supplementary Figs 1 and 2). For hypertension, the mediating effect was delineated by three significant indirect paths (indirect effect *P* < 0.05): Path 1—Hypertension → CSVD burden → PSMD → Categorization in MCOST (indirect effect value = −0.120), Path 2—Hypertension → PSMD → Categorization in MCOST (indirect effect value = −0.433) and Path 3—Hypertension → CSVD burden → Categorization in MCOST (indirect effect value = −0.501). For coronary heart disease, the mediating effect also comprised three indirect paths: Path 1—Coronary heart disease → CSVD burden → PSMD → Categorization in MCOST (indirect effect value = −0.172), Path 2—Coronary heart disease → PSMD → Categorization in MCOST (indirect effect value = −0.334) and Path 3—Coronary heart disease → CSVD burden → Categorization in MCOST (indirect effect value = −0.639). However, it is worth noting that only the indirect effect of Path 2 was significant (Fig. 5). After adjustment for age and sex, the findings were unchanged (Supplementary Fig. 3).

## Discussion

The current study revealed that PSMD mediated the relationship between all CSVD features (total burden, lacunes, CMBs, WMH and EPVS) and cognitive outcomes, including MMSE (global cognition) and MCOST Categorization (executive function). The results further indicate that hypertension and heart disease (vascular risk factors) could serve as upstream contributors to the CSVD–PSMD–cognitive impairment pathway.

A growing body of research has begun to clarify the mechanisms linking WM injury to cognitive impairment. Andrew J. Lawrence *et al*. revealed disturbances in brain network connectivity associated with CSVD, noting that network global efficiency either fully or partially mediates the relationship between MRI markers and cognitive performance.^38,39^ Yeo Jin Kim *et al*. identified that periventricular WM hyperintensities were implicated in gait disturbances, with mean FA serving as a mediator.^23^ Similarly, Pedro Henrique Rodrigues da Silva *et al*. found that the posterior thalamic radiation tract mediated the influence of WMH volume on processing speed scores.^24^ Moreover, emerging evidence suggests that deep medullary vein discontinuity may serve as a potential early neuroimaging biomarker for CSVD. Meng *et al.* proposed WMHV as a mediator linking deep medullary veins to cognitive decline.^40^ These findings align with ours, emphasizing the critical role of WM injury in the CSVD–cognitive impairments association. Further investigations on these pathways may provide valuable insights for diagnostic and therapeutic advancements.

WM in the brain is composed of highly organized networks that are crucial for neurobehavioural functions.^41^ CSVD encompasses a range of pathological conditions that affect the small vessels of the brain, potentially leading to a series of deleterious outcomes including chronic hypoperfusion and hypoxic stress, compromised blood–brain barrier integrity, disturbances in cerebral fluid drainage and vascular inflammation.^42,43^ The cumulative effect of these pathologies can result in focal or diffuse WM damage, characterized by demyelination and axonal loss. Such damage compromises the structural integrity of WM tracts, which are essential for facilitating efficient neural communication across various brain regions. Consequently, this disruption can lead to cognitive, neuropsychiatric and motor disturbances.^21^ Understanding the pathological mechanisms underlying CSVD is of critical importance to better address its wide-ranging impacts on brain function.

Expanding on previous investigations that assessed WM injuries using standard DTI markers and structural network metrics, our research used the novel marker of PSMD to investigate white matter injury’s mediating role between CSVD and cognitive impairment. The traditional methods have some shortcomings, including the propensity for inaccuracies in automated WMH measurement, data-intensive post-processing requirements and a relatively poor correlation with clinical symptoms. In contrast, PSMD provides a more sensitive and reliable measure of diffuse white matter integrity, detecting subtle changes that may missed by standard MRI metrics.^44^ An increase in PSMD reflects more extensive and severe microstructural degradation within WM. Such degradation likely indicates a broader spread of axonal damage and demyelination, both of which are characteristic features of severe CSVD. This extensive damage could potentially explain the cognitive impairments observed in patients with heavier CSVD burdens. Understanding the mediating roles of PSMD offers critical insights into the pathophysiology of CSVD-related cognitive decline. Moreover, our results indicate that PSMD could be a useful imaging marker for studies investigating cognitive injury associated with CSVD, requiring longitudinal validation.

PSMD allows for quick generation of the metric, which can be beneficial for patients in clinical settings, enabling timely initial screening and monitoring. Additionally, PSMD can potentially be a useful indicator of overall brain damage, which can be monitored over time through longitudinal assessments to track disease progression and treatment efficacy. Furthermore, PSMD shows consistent performance despite scanner and protocol differences, with high test–retest reliability observed. This consistency across different centres and hospitals can facilitate multi-centre research and improve the standardization of patient care.

Our study suggests that vascular factors, especially hypertension and heart disease, may serve as upstream determinants in the cascade linking CSVD, PSMD and cognitive impairment. Utilizing the UK Biobank data, Jun Shen *et al*. revealed that the association between vascular burden (including hypertension, hyperlipidaemia, diabetes, smoking and body mass index) and cognitive impairment was mediated by brain network efficiency.^22^ Moreover, Bonnie Yin Ka Lam revealed PSMD’s mediating role in four distinct relationships: hypertension-processing speed, diabetes-processing speed, smoking-processing speed and smoking-Montreal Cognitive Assessment memory domain associations.^34^ The findings presented by Shen *et al*. and Lam are partially consistent with our results. However, we did not observe a mediating effect of PSMD in the relationships between smoking, hyperlipidaemia, BMI, diabetes and cognitive function. This inconsistency could be explained by our restricted number of participants. Additionally, we employed a chain mediation analysis that incorporated both CSVD burden and PSMD, rather than the simple mediation analysis typically used in prior research. This methodological distinction could also contribute to inconsistencies between our findings and those of others. We will enrol more participants and undertake longitudinal studies in the future to deepen our understanding of the relationship among vascular risk factors, CSVD, WM injury, as well as cognition.

Several limitations of our study need to be acknowledged. First, the cross-sectional nature of the research design precludes definitive causal interpretations. There exists a need for future studies utilizing multivariate longitudinal approaches to better examine the dynamic relationship of CSVD, PSMD and cognition.^45^ Second, PSMD reflects overall white matter integrity but lacks specificity in differentiating between underlying pathological processes such as inflammation, axonal loss and demyelination. Further investigations should examine PSMD in multiple neurological conditions to clarify its biomarker characteristics in distinct pathological contexts. It is also important to explore PSMD in conjunction with other regional indicators to better capture small localized changes and improve its diagnostic and predictive value. Third, while the FMRIB58 template enables cross-study comparisons, its derivation from younger adults may introduce subtle biases when applied to aging populations with atrophy or vascular lesions. Future studies should explore the use of age-specific templates for improved alignment accuracy. Lastly, PSMD is heavily dependent on the registration and skeletonization steps and is sensitive to movement artifacts, which may affect the accuracy of our findings.

In conclusion, our study found that CSVD could impact cognitive function in older adults through the aggravated WM injury measured by PSMD. Hypertension and heart disease may act as upstream determinants in the pathway linking CSVD, PSMD and cognitive dysfunction. PSMD could be a useful imaging marker for investigating CSVD-related disorders in clinical practice. These findings warrant verification through more comprehensive future longitudinal research.

## Supplementary Material

fcaf233_Supplementary_Data

## Data Availability

The datasets are available from corresponding author upon reasonable request.
