# Supplementary material for: Peak width of skeletonized mean diffusivity mediates the relationship between cerebral small vessel disease burden and cognitive impairment in community-dwelling older adults
Source: Brain Commun. 2025 Jun 10;7(3):fcaf233. doi: 10.1093/braincomms/fcaf233 (PMC12203534; doi:10.1093/braincomms/fcaf233)
Supplement: fcaf233_Supplementary_Data [file fcaf233_supplementary_data.zip › Supplementary_material.docx]

**Supplementary Materials**

1. **Supplementary Methods**

**Neurological and neuropsychological assessments**

Each participant was administered a battery of neuropsychological tests to assess their global cognition, executive function, spatial construction function, memory, language, and attention. The battery contained the following:

1) Mini-Mental State Examination for global cognition. The MMSE is a concise tool used for evaluating cognitive function and screening for cognitive impairment. Developed in 1975 by Folstein et al^1^, it covers various cognitive domains including memory, attention, language, and spatial ability through a 30-point questionnaire. Tasks involve simple questions and commands to assess mental status, such as naming objects and following basic commands. Widely used in clinical and research settings, the MMSE helps in detecting and monitoring cognitive decline, especially in conditions like dementia and Alzheimer’s disease. Its brevity and comprehensiveness make it a standard assessment instrument globally.

2) The Conflicting Instructions Task, adapted from part of the Frontal Assessment Battery, was used to measure executive function32. First, the participants were asked to tap their fingers following the conflicting instructions (sensitivity to interference): "tap twice when I tap once, " and "tap once when I tap twice. " In addition, the participants were asked to do "Go/No-Go" (inhibitory control): "tap once when I tap once” and “do not tap when I tap twice. ".

3) The Stick Test, adapted from the Stick Construction Test, was used to measure spatial construction function and memory33. This 10-item test was first administered as a copying task. The participants were given 4 wooden sticks and asked to copy the examiner's model exactly. The participants were asked to recall and construct the previous pattern after copying the current one. After the 10 designs were copied, the participants were asked to construct the reverse pattern of the examiner's model.

4) The Modified Common Objects Sorting Test, adapted from the Object Sorting Test, was used to measure language and executive function34. The test material consisted of 42 pictures of common objects familiar to Chinese people. The participants were first required to name each object in the picture. Then, the participants were asked to sort all the objects into 7 different groups. The participants were then asked, "Why do all these belong together? ".

5) The Auditory Verbal Learning Test, adapted from the California Verbal Learning Test, was used to measure verbal memory of the participants35. This article is protected by copyright. All rights reserved. The participants were presented 12 words over 5 trials, followed by delayed recall and recognition trials.

6) Trail Making Tests A and B, adapted from a subtest of the Halstead–Reitan neuropsychological battery, were used to measure attention and executive function, including visual motor skills, fast visual search, and cognitive set-shifting. The participants were required to connect 25 consecutive targets with numbers inside squares or circles on a sheet of paper. There were 2 parts to the test: A, in which the targets were all numbers, and the participants needed to connect them in sequential order (e.g. 1, 2, 3); and B, in which the participants were asked to connect numbers in sequential order with the alternation of a square and circle (e.g. "1" in a square, "2" in a circle).

All tests were conducted in Chinese by study psychometrists within 90 minutes. Normative data of these tests have been reported elsewhere37. Impairment in a specific neuropsychological test was defined as having a test score > 1 standard deviation (SD) worse than the mean value in the corresponding age-specific groups.

1. **Supplementary Figures**

**
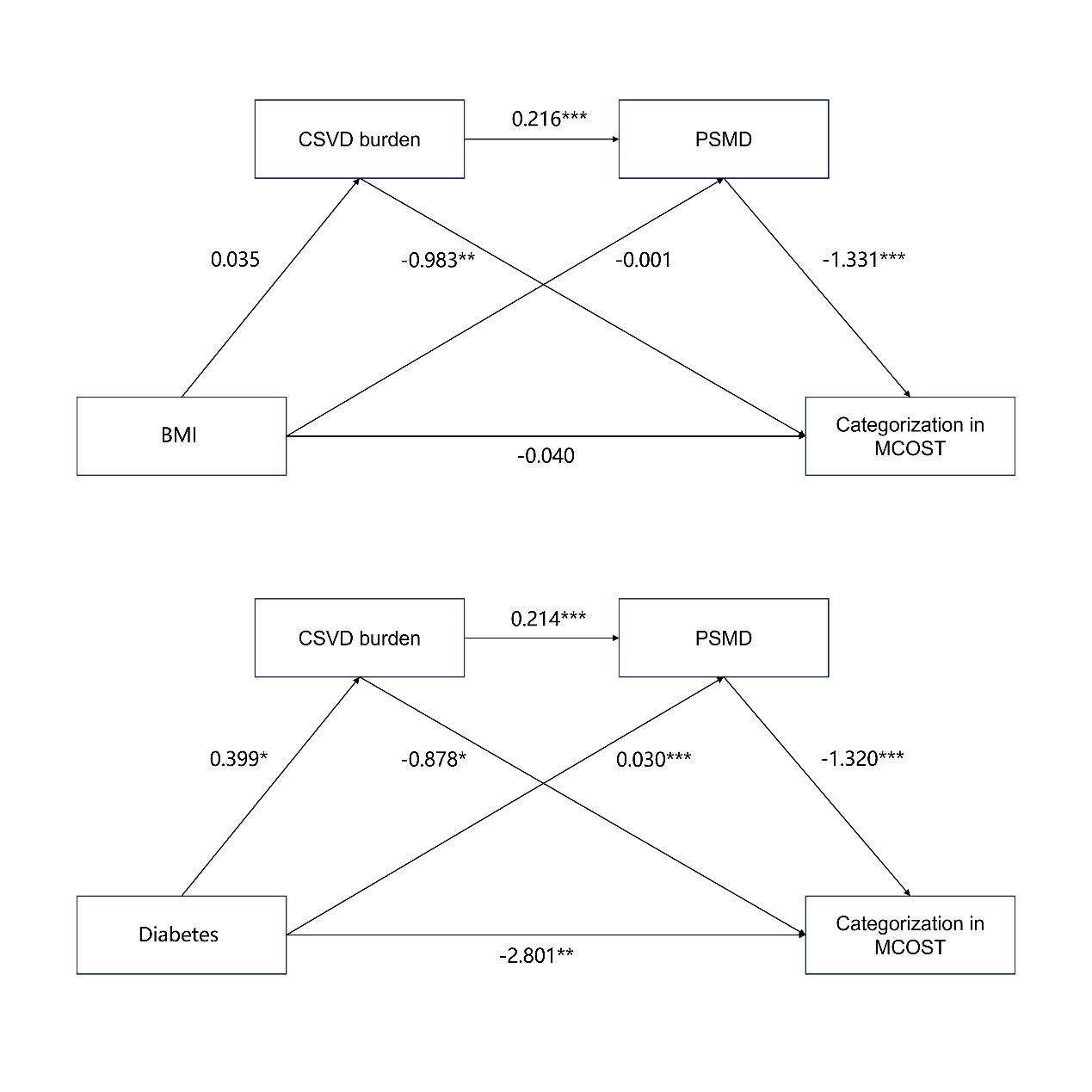
**

**Supplementary Figure 1: Influence of BMI and Diabetes on Cognitive Function via CSVD and PSMD Pathways.** Illustrating findings from an **exploratory chain mediation analysis using structural equation modeling** to assess how BMI and diabetes indirectly affect cognitive function through CSVD and PSMD. The total number of participants was 271. Standardized coefficients (β) for each pathway are reported, with significance levels highlighted as *P<0.05, **P<0.01, ***P<0. 001. BMI, Body mass index; CSVD, Cerebral small vessel disease; PSMD, Peak width of skeletonized mean diffusivity; MCOST, Modified common objects sorting test.


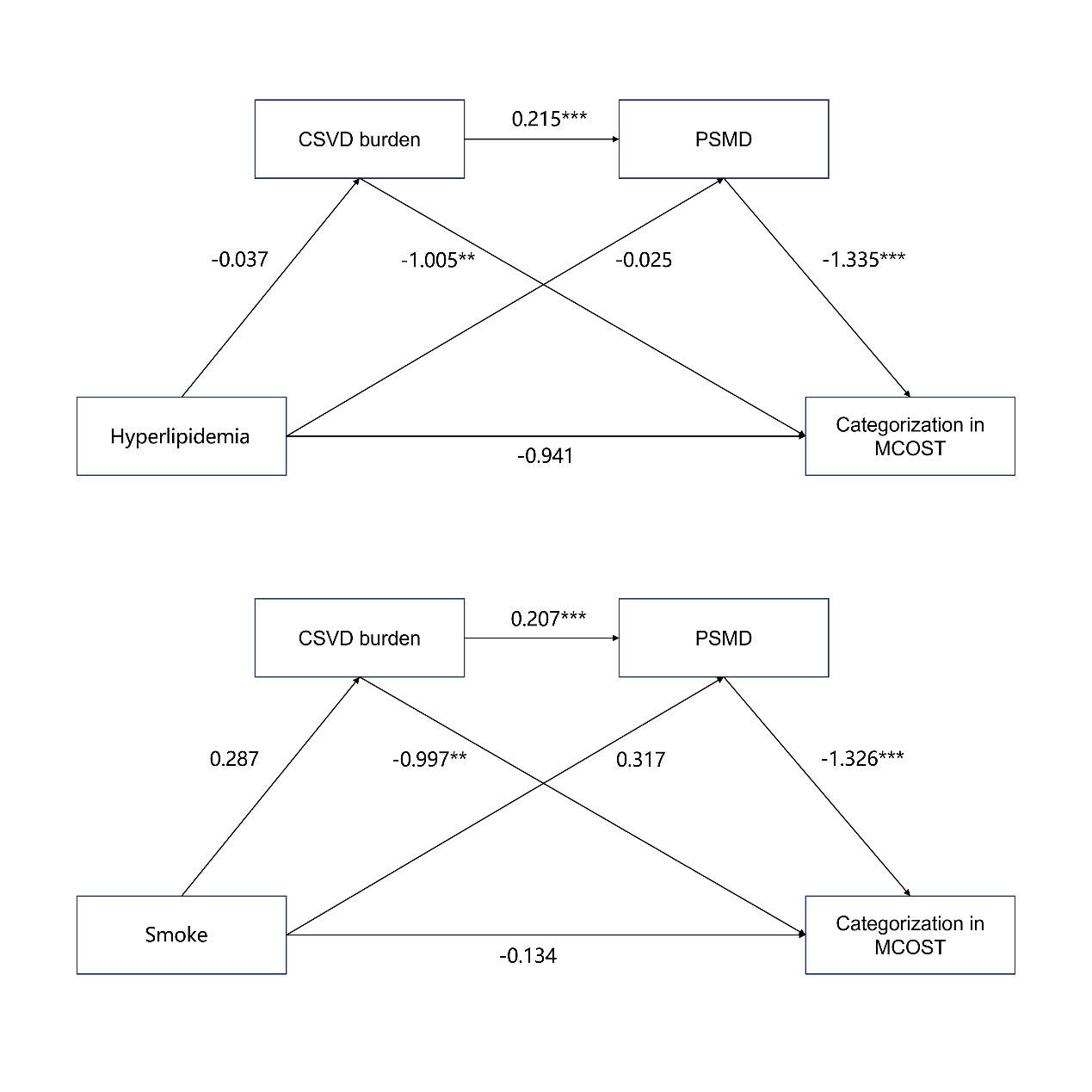


**Supplementary Figure 2: Influence of Hyperlipidemia and Smoke on Cognitive Function via CSVD and PSMD Pathways.** Illustrating findings from an **exploratory chain mediation analysis using structural equation modeling** to assess how hyperlipidemia and smoke indirectly affect cognitive function through CSVD and PSMD. The total number of participants was 271. Standardized coefficients (β) for each pathway are reported, with significance levels highlighted as *P<0.05, **P<0.01, ***P<0. 001.CSVD, Cerebral small vessel disease; PSMD, Peak Width of Skeletonized Mean Diffusivity; MCOST, Modified Common Objects Sorting Test.


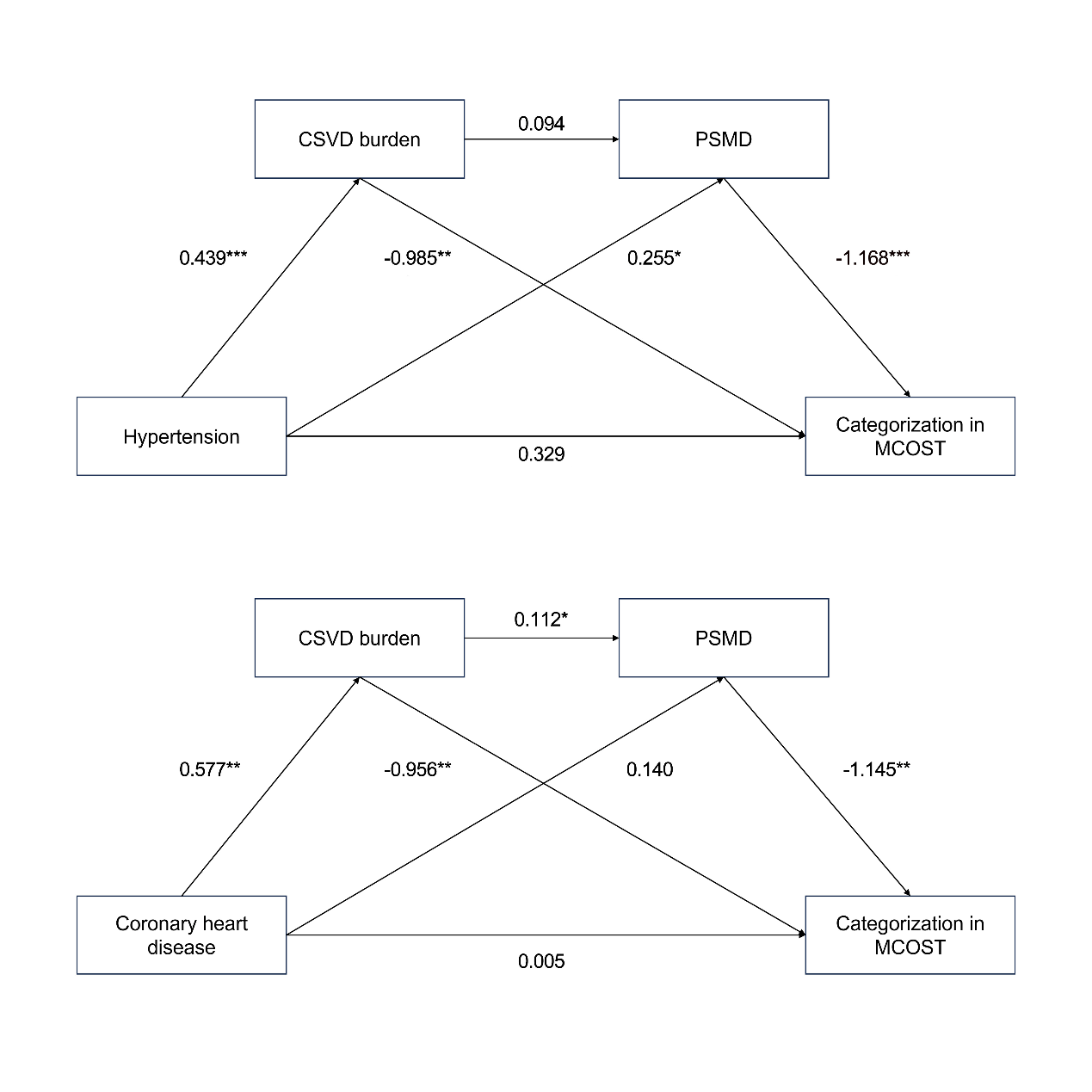


**Supplementary Figure 3:** **Influence of Hypertension and Coronary Heart Disease on Cognitive Function via CSVD and PSMD Pathways with age and sex as covariates.** Illustrating findings from an exploratory **chain mediation analysis using structural equation modeling** to assess how hypertension and coronary heart disease indirectly affect cognitive function through CSVD and PSMD. The total number of participants was 271. Standardized coefficients (β) for each pathway are reported, with significance levels highlighted as *P<0.05, **P<0.01, ***P<0. 001.CSVD, Cerebral small vessel disease; PSMD, Peak width of skeletonized mean diffusivity; MCOST, Modified common objects sorting.

1. **Supplementary Tables**

**Supplementary Table1 Association between total CSVD burden and domain-specific cognition.**

|  | **β** | ***P* value** | **Std. Error** |
| --- | --- | --- | --- |
| **Conflicting Instructions Task** |  |  |  |
| Conflicting correct tapping | 0.076 | 0.227 | 0.093 |
| Go/ No-Go correct tapping | -0.072 | 0.232 | **0.009** |
| **Stick Test** |  |  |  |
| Imitate | -0.141 | 0.018 | **0.031** |
| Recall | -0.073 | 0.228 | 0.058 |
| Rotate | -0.041 | 0.477 | 0.067 |

Note: Model adjust for age, sex, education levels, and APOE Ɛ4 carrier status. Significant p values are in bold. CSVD, Cerebral small vessel disease.

**Supplementary Table2 Association between WMHV and domain-specific cognition.**

|  | **β** | **Std. Error** | ***P* value** |
| --- | --- | --- | --- |
| **Conflicting Instructions Task** |  |  |  |
| Conflicting correct tapping | 0.134 | 0.009 | **0.033** |
| Go/ No-Go correct tapping | -0.031 | 0.001 | 0.610 |
| **Stick Test** |  |  |  |
| Imitate | 0.021 | 0.003 | 0.722 |
| Recall | -0.158 | 0.006 | **0.009** |
| Rotate | -0.097 | 0.007 | 0.090 |

Note: Model adjust for age, sex, education levels, and APOE Ɛ4 carrier status. Significant p values are in bold. WMHv, White matter hyperintensity volume; APOE Ɛ4, Apolipoprotein E ε4.

**Supplementary Table3 Association between Lacune and domain-specific cognition.**

|  | **β** | **Std. Error** | ***P* value** |
| --- | --- | --- | --- |
| **Conflicting Instructions Task** |  |  |  |
| Conflicting correct tapping | -0.111 | 0.078 | 0.073 |
| Go/ No-Go correct tapping | -0.152 | 0.007 | **0.010** |
| **Stick Test** |  |  |  |
| Imitate | -0.227 | 0.028 | **＜0.001** |
| Recall | -0.039 | 0.054 | 0.520 |
| Rotate | -0.010 | 0.063 | 0.863 |

Note: Model adjust for age, sex, education levels, and APOE Ɛ4 carrier status. Significant p values are in bold.

**Supplementary Table4 Association between CMBs and domain-specific cognition.**

|  | **β** | **Std. Error** | ***P* value** |
| --- | --- | --- | --- |
| **Conflicting Instructions Task** |  |  |  |
| Conflicting correct tapping | -0.021 | 0.017 | 0.735 |
| Go/ No-Go correct tapping | -0.195 | 0.002 | **＜0.001** |
| **Stick Test** |  |  |  |
| Imitate | -0.306 | 0.007 | **＜0.001** |
| Recall | -0.160 | 0.013 | **0.007** |
| Rotate | -0.049 | 0.016 | 0.384 |

Note: Model adjust for age, sex, education levels, and APOE Ɛ4 carrier status. Significant p values are in bold. CMBs, Cerebral microbleeds.

**Supplementary Table5 Association between EPVS and domain-specific cognition.**

|  | **β** | **Std. Error** | ***P* value** |
| --- | --- | --- | --- |
| **Conflicting Instructions Task** |  |  |  |
| Conflicting correct tapping | -0.037 | 0.113 | 0.557 |
| Go/ No-Go correct tapping | -0.082 | 0.011 | 0.175 |
| **Stick Test** |  |  |  |
| Imitate | -0.047 | 0.037 | 0.436 |
| Recall | -0.110 | 0.069 | 0.073 |
| Rotate | -0.053 | 0.081 | 0.358 |

Note: Model adjust for age, sex, education levels, and APOE Ɛ4 carrier status. Significant p values are in bold. EPVS, Enlarged perivascular space.

**Supplementary Table6 Association between PSMD and domain-specific cognition.**

|  | **β** | **Std. Error** | ***P* value** |
| --- | --- | --- | --- |
| **Conflicting Instructions Task** |  |  |  |
| Conflicting correct tapping | 0.097 | 0.105 | 0.171 |
| Go/ No-Go correct tapping | 0.020 | 0.010 | 0.767 |
| **Stick Test** |  |  |  |
| Imitate | -0.244 | 0.035 | **＜0.001** |
| Recall | -0.094 | 0.067 | 0.173 |
| Rotate | -0.110 | 0.078 | 0.092 |

Note: Model adjust for age, sex, education levels, and APOE Ɛ4 carrier status. Significant p values are in bold. PSMD, Peak Width of Skeletonized Mean Diffusivity.

|  | Mean | Std Error | 95% CI Lower | 95% CI Upper |
| --- | --- | --- | --- | --- |
| MMSE score | | | | |
| Group1 | -0.62 | 0.21 | -1.02 | -0.22 |
| Group2 | -1.09 | 0.41 | -1.84 | -0.24 |
| Categorization in MCOST score | | | | |
| Group1 | 0.04 | 0.62 | -1.63 | 0.82 |
| Group2 | 0.05 | 1.73 | -6.77 | -0.11 |

**Supplementary Table7 Interactive Effects of PSMD and CSVD Burden on Cognitive Performance Using Bootstrap Analysis**

**Supplementary Reference**

1. Folstein MF, Folstein SE, McHugh PR. "Mini-mental state". A practical method for grading the cognitive state of patients for the clinician. Journal of psychiatric research. 1975;12(3):189-198.
